# Risk factors for community-acquired pneumonia among adults in Kenya: a case–control study

**DOI:** 10.1186/s41479-017-0041-2

**Published:** 2017-11-25

**Authors:** Esther Muthumbi, Brett S. Lowe, Cyprian Muyodi, Esther Getambu, Fergus Gleeson, J. Anthony G. Scott

**Affiliations:** 1 KEMRI-Wellcome Trust Research Programme, Center for Geographical Medicine Research Coast, Kilifi, Kenya; 2 Centre for Tropical Medicine & Global Health, University of Oxford, Oxford, UK; 3 Coast Provincial General Hospital, Mombasa, Kenya; 4 Department of Radiology, Churchill Hospital, University of Oxford, Oxford, UK; 5 Department of Infectious Disease Epidemiology, London School of Hygiene & Tropical Medicine, London, UK

**Keywords:** Community acquired pneumonia, Adults, Africa, Risk factors, Air pollution

## Abstract

**Background:**

Pneumonia is a leading cause of morbidity and mortality among adults worldwide; however, the risk factors for community-acquired pneumonia in Africa are not well characterized.

**Methods:**

The authors recruited 281 cases of community-acquired pneumonia and 1202 hospital controls among patients aged ≥15 years who attended Kilifi District Hospital/Coast Provincial General Hospital in Kenya between 1994 and 6. Cases were admissions with an acute illness with ≥2 respiratory signs and evidence of consolidation on a chest radiograph. Controls were patients without signs of pneumonia, frequency matched by age, sex and hospital. Risk factors related to socio-demographic factors, drug use, clinical history, contact patterns and exposures to indoor air pollution were investigated by questionnaire, anthropometric measurements and laboratory assays. Associations were evaluated using a hierarchical logistic regression model.

**Results:**

Pneumonia was associated with human immunodeficiency virus (HIV) infection (Odds Ratio [OR] 2.06, 95% CI 1.44–3.08), anemia (OR 1.91, 1.31–2.74), splenomegaly (OR 2.04, 95% CI 1.14–3.41), recent history of pneumonia (OR 4.65, 95% CI 1.66–12.5), history of pneumonia >2 years previously (OR 17.13, 95% CI 5.01–60.26), coryza in the 2 weeks preceding hospitalization (OR 2.09, 95% CI 1.44–3.03), current smoking (2.19, 95% CI 1.39–3.70), use of khat (OR 3.44, 95% CI 1.72–7.15), use of snuff (OR 2.67, 95% CI 1.35–5.49) and contact with several animal species. Presence of a Bacillus Calmette-Guerin (BCG) scar was associated with protection (OR 0.51, 95% CI 0.32–0.82). The risk factors varied significantly by sex.

**Conclusion:**

Pneumonia in Kenyan adults was associated with global risk factors, such as HIV and smoking, but also with specific local factors like drug use and contact with animals. Intervention strategies should account for sex-specific differences in risk factors.

**Electronic supplementary material:**

The online version of this article (10.1186/s41479-017-0041-2) contains supplementary material, which is available to authorized users.

## Background

Community-acquired pneumonia (CAP) is a common cause of morbidity and mortality worldwide. In Africa, CAP is associated with an in-hospital mortality of 6–15% among adults, as reported from hospital-based studies [1–3].

Studies from high-income countries have identified several risk factors for CAP including smoking [4–7], age > 65 years [4, 8, 9], immunosuppression by any cause [8], underlying lung diseases such as chronic obstructive pulmonary disease (COPD) [7–10], recent viral upper respiratory infections (URTI) [11–13] and the presence of co-morbidities [6]. In addition, low body-mass index (BMI), contact with children and poor dental hygiene were identified as risk factors for CAP in a systematic literature review and meta-analysis from studies in Europe [5].

Different environmental and socio-economic circumstances in sub-Saharan Africa are likely to give rise to different risks for pneumonia, including younger age at presentation, which has been observed [14]. There is a single published study of pneumonia risk factors in tropical Africa, which is confined to human immunodeficiency virus (HIV)-infected adults in Kenya. This identified additional risk factors as being single, widowed or divorced (i.e. not married), being of low socio-economic status and experiencing overcrowding in the home [15]. Here the authors present an analysis of a previously unpublished dataset from 1994 to 1996, exploring the risk factors for pneumonia among adults in Kenya.

## Methods

### Study design

This case–control study was conducted at Kilifi District Hospital (KDH), and Coast Provincial General Hospital (CPGH) in Kenya among patients who presented either to the outpatient clinic or the casualty department. Cases were adult patients aged ≥15 years who were admitted with CAP. CAP was defined as an acute illness (<14 days), characterized by at least two respiratory symptoms (cough, sputum, breathlessness, chest pain, hemoptysis or fever) and evidence of consolidation on a chest radiograph. The radiographs were read by a study physician and later confirmed, independently, by a consultant thoracic radiologist.

Controls were adults aged ≥15 years who presented to the outpatient clinics of the same hospitals who did not meet the clinical case definition of CAP. They were frequency matched on age, sex and hospital of presentation in a ratio of 4:1 with cases. Controls with diagnoses that were strongly associated with the exposures of interest (e.g. meningitis, septicemia, tuberculosis, Kaposi’s sarcoma, sickle cell crisis, diabetic ketoacidosis or oropharyngeal candidiasis) were excluded.

A target sample size of 1500 (300 cases: 1200 controls) was sought for the study. The study was powered to detect relevant odds ratios using a range of prevalence estimates for a wide variety of exposures included in the study (Additional file 1).

### Study procedures

Patients were questioned on clinical history, lifestyle habits and contact history using a standard questionnaire. Anthropometry and a physical examination were performed for all participants in a standardized manner. Venous blood was collected and tested for HIV-1 antibodies by enzyme-linked immunosorbent assay (ELISA), malaria parasites by microscopy, and sickle cell status. A full hemogram, glycosylated hemoglobin (HbA1C) test and blood grouping (ABO) were also performed.

### Statistical analysis

Associations with case status were analyzed using logistic regression, adjusting for the matching variables (age, sex and route of presentation) in each model. Because this was the first study of risk factors for pneumonia in an unselected population in Africa, a wide range of potential exposures were explored. A hierarchical process was used to define the final model, whereby related exposures were first examined in intermediary models before the best representative exposures were selected for a final, multi-variable model. The intermediary models examined 5 categories of related variables (Table 1). Within each category, univariate analysis was performed and variables were selected with a likelihood ratio (LR) *p*-value of <0.1 to include in the intermediary multivariable model. Risk factors contributing significantly (LR test, *p* = <0.05) to the intermediary multivariable models were subsequently included in a final multivariable model. Backward stepwise analysis was used in each of the multivariable analyses. In addition, prompted by the presence of sex-specific exposures (e.g. pregnancy) and several instances of effect modification due to sex, two sex-restricted models were developed following a similar hierarchical process.

**Table 1:** Variable categories for the intermediary logistic regression models

Group name	Variables
Socio-demographic variables	Ethnic group, religion, recent immigration, years of education, monthly income, type of house and employment status
Contact variables	Number of children in the household, contact with children of different ages, with or without an URTI, number of adults in the household, size of house, hours of childcare, sharing a room with children when sleeping, contact with a case of pneumonia, recent travel beyond the immediate residential vicinity, use of public minibuses for transport (matatu), frequenting social places and contact with selected animals, working and living in different locations
Drug use	Quantified alcohol consumption and use of traditional brews (matingas, busaa, changaa, mnazi, muratina), quantified present and past cigarette smoking, use of ground tobacco (snuff), use of Khat (miraa).
Indoor air pollution	Exposure to cooking fuel smoke in the home, use of mosquito coils, ventilation in the cooking room, use of air conditioning, passive smoking and occupational exposure during welding
Clinical variables	Chronic bronchitis, recent history of viral URTI (<14 days), previous history of pulmonary TB, previous pneumonia, body mass index, mid upper-arm circumference, anemia, splenomegaly, malaria parasitemia, sickle cell status, HIV sero-positivity, ABO blood group, Glycosylated hemoglobin (HbA1c) and current pregnancy status.

HIV, human immunodeficiency virus; TB, tuberculosis; URTI, upper respiratory tract infection

Some variables, e.g. BMI and presence of a scar following Bacillus Calmette-Guerin (BCG) vaccine, were introduced shortly after the study had begun, leading to some missing data (Additional file 1). However, this is unlikely to have caused bias unless there was a systematic difference in the admissions across the time periods of the study; therefore, data imputation was not used.

All statistical analysis were performed using STATA V.13 (Stata Corp, College Station, Texas, United States [US]).

## Results

Between March 1994 and May 1996, 301 cases and 1202 controls were recruited. After review of the chest radiographs by the radiologist, 20 of the original cases were excluded as non-CAP. Among the 281 remaining cases, 177 (63%) were male and 22 (7.8%) were aged ≥55 years. The matching achieved a similar distribution of age, sex and route of recruitment across cases and controls (Table 2). Among controls, malaria was the most common presenting diagnosis, accounting for 26% (316/1202, Additional file 1: Table S3).

**Table 2:** Distribution of matching variables across cases and controls

Matching variable	Categories	Cases	%	Controls	%
Sex	Male	177	63%	749	62%
	Female	104	37%	453	38%
Age group	15–24	65	23%	267	22%
	25–29	48	17%	265	22%
	30–34	63	22%	193	16%
	35–44	61	22%	255	21%
	45–54	22	8%	110	9%
Route of recruitment	CPGH Filter clinic	45	16%	214	18%
	CPGH Casualty	188	67%	785	65%
	Kilifi District Hospital	48	17%	203	17%

The results of the univariate analysis for 72 variables, by category of exposure, are listed in the Additional file 1: Tables S4–S8. In Table 3 the results of the intermediary and final adjusted models are presented for 25 variables, with significant results at the intermediary model stage.

**Table 3:** Risk factors for pneumonia in intermediate and final regression models

	Observed results				Intermediate models		Final model		
Variable	Control	%	Case	%	aOR^a^	95% CI	aOR^b^	95% CI	*p*-value
*Clinical*									
HIV infection	356	30.3	147	52.3	2.13	(1.48, 3.07)	2.06	(1.44,3.08)	<0.001
History of coryza	283	23.5	120	43.2	2.3	(1.62,3.26)	2.09	(1.44,3.03)	<0.001
Splenomegaly	81	6.8	46	17.2	2.34	(1.44,3.81)	2.04	(1.17,3.41)	0.009
Anaemia	607	51.1	202	72.4	1.88	(1.31,2.68)	1.91	(1.31,2.74)	0.001
Malaria	155	13.2	9	3.2	0.17	(0.08,0.36)	0.12	(0.06,0.29)	<0.001
Presence of BCG scar	1030	88	207	81.5	0.58	(0.37,0.90)	0.51	(0.32,0.82)	0.005
History of previous pneumonia									
None	1180	98.3	248	89.21	1		1		<0.001
> 2 yrs. ago	4	0.3	17	6.1	20.39	(5.08, 81.79)	17.13	(5.01, 60.26)	
< 2 yrs. ago	16	1.3	13	4.7	5.26	(1.86, 14.84)	4.65	(1.66, 12.54)	
HbA1C									
< 4.0	677	60.2	141	52.4	0.7	(0.64, 1.69)	–		
4.0–5.6	397	35.3	118	43.4	1		–		
5.7–6.4	29	2.6	3	1.1	0.27	(0.07, 1.00)	–		
> 6.5	21	1.9	7	2.6	1.15	(0.35, 3.72)	–		
MUAC									
< 22	200	16.8	65	24.1	1		1		<0.001
22–23	179	15	86	31.9	1.45	(0.99, 2.13)	1.92	(1.19, 3.11)	
24–25	239	20.1	65	24.1	0.8	(0.53, 1.20)	1.02	(0.62, 1.67)	
26–27	292	24.5	34	12.6	0.34	(0.21, 0.53)	0.53	(0.30, 0.94)	
28+	281	23.6	20	7.4	0.21	(0.12, 0.35)	0.38	(0.20, 0.72)	
*Socio-demographic*									
Level of education									
None	221	18.4	72	25.9	1				
1–6 years	244	20.3	63	22.7	0.61	(0.40, 0.93)	–		
Primary	313	26	65	23.4	0.44	(0.28, 0.68)	–		
Secondary	310	25.8	64	23	0.41	(0.26, 0.65)	–		
Tertiary	114	9.5	14	5	0.26	(0.13, 0.51)	–		
Employment Status									
Unemployed	423	35.2	86	30.9	1				
Employed	715	59.5	187	67.3	1.47	(1.05, 2.05)	–		
In Education	64	5.3	5	1.8	0.43	(0.16, 1.14)	–		
*Air pollution*									
More than one ventilations in cooking room^c^ (*n* = 1036)	812	78.4	171	70.1	0.61	(0.44, 0.85)	–		
*Drugs*									
Smoking									
None smoker	697	58	117	42.1	1				<0.001
Passive smoker	177	14.7	31	11.2	0.92	(0.59, 1.44)	0.86	(0.51, 1.57)	
Ex-smoker	88	7.3	31	11.2	2.9	(1.76, 4.77)	2.44	(1.38, 4.72)	
Recent ex-smoker	12	1	12	4.3	7.57	(3.19, 17.98)	10.4	(3.49, 35.38)	
Current smoker	228	19	87	31.3	2.24	(1.48, 3.40)	2.19	(1.39, 3.70)	
Smoking pack years									
0	885	80.3	153	65.1	1				
1–5	113	10.3	36	15.3	1.82	(1.11, 2.99)	–		
6–10	50	4.5	22	9.4	2.11	(1.11, 4.01)	–		
11–15	28	2.5	8	3.4	1.47	(0.60, 3.62)	–		
16–20	11	1	11	4.7	4.53	(1.73, 11.83)	–		
20+	15	1.4	5	2.1	2.16	(0.71, 6.58)	–		
Khat	42	3.5	31	11.2	2.42	(1.40, 4.18)	3.44	(1.72, 7.15)	<0.001
Snuff	48	4	23	8.3	2.79	(1.57, 4.99)	2.67	(1.35, 5.49)	0.005
Muratina	2	0.2	4	1.4	8.61	(1.42, 52.2)	–		
Alcohol	218	18.1	84	30.2	1.46	(1.01, 2.08)	–		
*Contacts*									
Work and lives in different locations	317	26.4	87	31.3	1.49	(1.08, 2.07)	1.66	(1.13, 2.57)	0.011
Visited a disco in the last 2 weeks	36	3	3	1.1	0.29	(0.08, 0.97)	0.16	(0.05, 0.81)	0.024
Visited a café in last 2 weeks	131	10.9	53	19.1	2.31	(1.57, 3.40)	1.82	(1.08, 2.85)	0.024
Used a matatu in last 2 weeks	947	78.9	199	71.6	0.59	(0.43, 0.81)	0.46	(0.31, 0.68)	<0.001
Exposure to ducks	12	1	9	3.2	3.03	(1.18, 7.78)	4.14	(1.28, 13.46)	0.018
Exposure to goats	219	18.2	76	27.3	1.56	(1.09, 2.23)	1.69	(1.16, 2.59)	0.007
Exposure to chickens	471	39.2	143	51.4	1.48	(1.08, 2.03)	–		
Exposure to monkeys	3	0.2	5	1.8	8.73	(1.92, 39.63)	–		

OR = Odds Ratio; aOR = adjusted odds ratio;

^a^first stage multivariable model adjusted results (within-category)

^b^second stage multivariable model adjusted results

^c^among those cooking indoors

### Clinical factors

Previous history of pneumonia was a major risk factor for current pneumonia. This risk was almost 20-fold higher among those whose history of pneumonia was more than 2 years ago (Table 3). History of previous URTI, HIV infection, splenomegaly and anemia were all associated with a 2-fold increase in risk of pneumonia; HIV was present in 30% (356/1202) of the controls. Malaria and presence of a BCG scar was associated with a reduced risk of pneumonia. The risk of pneumonia was reduced by 35% for every cm increase in mid-upper arm circumference (MUAC, Table 3, Additional file 1: Table S4). For most of the significant clinical variables, there was no evidence of confounding by variables in other categories as the effect sizes varied little between intermediary and final models. Of note from the univariate analyses, there was no association between pneumonia and sickle cell trait, Blood group A or history of previous tuberculosis (Additional file 1: Table S4).

### Socio-demographic factors

In the intermediate model, pneumonia was inversely associated with the number of years in education and with current employment. However, these associations did not remain significant in the final model. In the univariate analysis, there was no evidence of an association between pneumonia and ethnicity, religion or economic status measured either as amount of income or type of roofing (Additional file 1: Table S5).

### Air pollution and related factors

Among a wide range of air-pollution variables, only two were significant in the univariate analyses; cooking in a room with only one ventilation exit (the door) was more common among cases; cooking for oneself was more common among controls (Additional file 1: Table S6). Pneumonia was not associated with indoor cooking, nor sleeping in the cooking room, nor with the type of fuel used for cooking.

### Drug use and related factors

The prevalence of drug use differed by sex. Males accounted for 87% (268/307) of alcohol consumers and 97% (312/321) of current smokers. Females were the largest consumers of snuff (60% vs. 40% in males), but did not report consumption of *busaa* and *matingas* (traditional brews). Current smokers and ex-smokers had a 2-fold increased risk of pneumonia compared to never-smokers; recent ex-smokers had a 10-fold increase in risk (Table 3). There was no evidence of increased risk with an increase in smoking pack-years. Passive smoking and alcohol intake were not associated with pneumonia.

### Contact patterns

Exposure to animals was reported in 43% (519/1202) of the controls. Significant associations in the intermediary model included exposure to monkeys, chickens, ducks and goats, of which only the latter two remained significant in the final model (Table 3). Adults frequenting cafés or working at a different location from home were at slightly increased risk of pneumonia; those using public minibuses (matatus) or visiting nightclubs had lower risks of pneumonia. In the univariate analysis, pneumonia was associated with the total number of other people in the home, and with the number of co-resident girls aged <5y. Contact with a child under the age of 5 years with coryza or sleeping in the same room as a child were not associated with pneumonia.

### Risk factors by sex

After restricting the analysis by sex, HIV infection, history of coryza, splenomegaly and anemia were independent risk factors shared by both sexes (Table 4). Decreasing MUAC, history of tuberculosis, smoking, use of khat and exposure to chickens were risk factors unique to males. Among females, use of snuff and exposure to ducks and sheep were unique risk factors. The presence of a BCG scar was a protective factor among males only.

**Table 4:** Risk factors for pneumonia by sex

	MALE			FEMALE		
Variable	aOR	95% CI	*p*-value	aOR	95% CI	*p*-value
*Clinical*						
HIV-infected	2.67	(1.51, 4.71)	0.001	3.06	(1.66, 5.64)	<0.001
History of coryza	2.36	(1.42, 3.92)	0.001	2.30	(1.26, 4.18)	0.006
Splenomegaly	2.24	(1.07, 4.72)	0.033	3.44	(1.55, 7.64)	0.002
Anemia	2.32	(1.34, 4.00)	0.003	3.25	(1.70, 6.20)	<0.001
Malaria	0.04	(0.01, 0.16)	<0.001	0.15	(0.05, 0.48)	0.001
Presence of BCG scar	0.33	(0.16, 0.69)	0.003			
Previous history of pneumonia						
Pneumonia >2 yrs. ago	8.35	(1.07, 65.25)	<0.001	26.57	(4.80, 147.21)	<0.001
Pneumonia <2 yrs. ago	17.99	(3.77, 85.73)		3.19	(0.63, 16.18)	
Previous history of TB infection						
TB >2 yrs. ago	4.38	(1.18, 16.31)	0.033	–		
TB <2 yrs. ago	7.51	(0.72, 77.95)		–		
MUAC						
< 22	1		<0.001	–		
22–23	2.60	(1.26, 5.36)		–		
24–25	0.94	(0.44, 2.00)				
26–27	0.47	(0.20, 1.10)		–		
28+	0.18	(0.07, 0.48)		–		
*Drugs*						
Smoking			0.001			
Passive smoker	2.12	(0.68, 6.68)		–		
Ex-smoker	3.00	(1.37, 6.54)		–		
Recent ex-smoker	13.06	(2.86, 59.58)		–		
Current smoker	2.79	(1.57, 4.98)		–		
Khat (miraa)	4.85	(2.07, 11.38)	<0.001	–		
Snuff	–			4.34	(1.87, 10.05)	0.001
*Contact*						
Exposure to chickens	2.47	(1.46, 4.17)	0.001			
Exposure to ducks	–			7.59	(1.47, 39.16)	0.015
Exposure to sheep	–			6.91	(1.02, 46.69)	0.047

HIV, human immunodeficiency virus; MUAC, middle upper arm circumference; TB, tuberculosis

## Discussion

These results suggest that several of the risk factors for pneumonia are common to both developed and developing countries. These include smoking tobacco, exposure to animals, recent URTI and anemia. The results have also identified novel modifiable risk factors, such as the use of snuff (ground tobacco) and khat, which are particular to this population.

Smoking is a well-established risk factor for CAP [7, 16]. The risk was highest among recent ex-smokers; this was interpreted to mean that their decision to stop smoking was influenced by an ailing respiratory system—a form of reverse causality. Passive smoking was not associated with an increased risk of pneumonia. Passive cigarette smoke exposure is a known risk factor for lower respiratory tract infections (LRTIs) among children [16–18], but not among adults.

Use of snuff and khat are novel associations found among women and men, respectively. Snuff use is a risk factor for oral cancer [19], while khat, a natural amphetamine, is associated with a range of effects from tooth decay to psychosis [20–22]. Khat, *Catha edulis* Forsk, is a shrub whose leaves and twigs are chewed for their stimulant effect and there is no obvious biological explanation of this association. Overall, however, drug use appeared to be a major avoidable risk factor.

History of coryza (representing URTIs) was associated with pneumonia [11]. Viral URTIs suppress immune function leading to increased susceptibility to secondary infections. Influenza vaccine can reduce both primary pneumonia and secondary bacterial pneumonia [23–26], and may be useful to prevent CAP in adults.

Patients with a previous history of pneumonia were at a higher risk of CAP in the current study, especially those whose initial episode occurred more than 2 years previously; in previous studies the risk was higher for more recent episodes [7]. Childhood pneumonia is associated with development of chronic lung disease and later hospitalizations with pneumonia [27–29] and in this study, 40% of cases were <30 years of age. In addition, patients with recent episodes of pneumonia are more likely to recognize the symptoms and the seriousness of the illness, and therefore more likely to seek early treatment, averting hospital admission. In Kenya, pneumococcal vaccines are rarely used in adults; however, post-hospitalization vaccination could potentially reduce the rate of recurrence with pneumonia [30].

The authors found that low social economic status (SES), measured directly by level of income and indirectly by proxy measures like the materials used for house construction, was not associated with pneumonia. However, higher education, increased use of *matatus* (minibuses for public transport) and frequent visits to a nightclub, which are all likely to be indicators of higher SES, were associated with a reduced risk of disease despite also implying more human contact and therefore, potentially, a greater risk of infection.

Indoor air pollution is a leading risk factor for respiratory diseases [31], including pneumonia, in children [32–34] though evidence in adults is weak [35]. In this study, exposure to air pollution, including biomass fuels, was not associated with an increased risk of pneumonia. However, this study relied upon questionnaire methods to ascertain exposure to air pollution and this is likely to admit significant misclassification. More direct measurements of air quality would be useful to estimate the role of this risk factor [36].

As in other studies from developed countries [37, 38], HIV infection was associated with a 2-fold increase in risk of CAP. This effect size is likely to be an under-estimate due to the use of hospital controls, despite attempts to exclude from the control population those with HIV-related diseases. The prevalence of HIV among controls was 30.3%; population-based estimates of HIV sero-prevalence at the same time were 7.5% [39]. Use of hospital controls also explains the seemingly protective effect of malaria infection. Infection with malaria parasites in the tropics is a common cause of presentation to hospitals with non-pneumonia syndromes. Interestingly, markers of chronic malaria infection, anemia and splenomegaly were strongly associated with pneumonia, suggesting that chronic or recurrent malaria may in fact be a risk factor for pneumonia, although splenomegaly may also be a marker of HIV or tuberculosis, both common in this population.

Evidence of BCG vaccination was associated with a 70% reduction in risk of pneumonia among men. The effect in women was smaller and non-significant. In the complementary study of pneumonia etiology conducted in the same cases, *Mycobacterium tuberculosis* was found in 9% [1]. Several vaccines have demonstrated non-specific protective and harmful effects, which differ by sex [40, 41]. For example, those who develop a BCG scar following vaccination are known to be less likely to get sepsis, with the beneficial effect occurring predominantly in girls [42]. These benefits have been observed to extend into adulthood [43]. The WHO suggests these heterologous effects of vaccines are intriguing, currently inexplicable and “warrant further research” [44].

The limitation of this study is that it is an analysis of historical data and therefore, due to changes in epidemiology in the ensuing years, the relative importance of some of the observations may have changed. First, several risk factors that were identified have changed in prevalence; for example, HIV-infection and malaria have both declined and smoking has increased. While this alters the public health importance of these factors and invalidates any attempt to calculate the current population-attributable fraction for pneumonia, it does not invalidate the etiological association.

Second, temporal changes may have modified the effect of risk factors; for example, the introduction of anti-retroviral therapy reduces the risk of pneumonia in HIV-infected individuals [45, 46]. Third, changes in urbanization and lifestyle habits may have introduced new risk factors for pneumonia, which are not captured by the dataset that has been analyzed. Nonetheless, historical data such as these can offer unique insights into the epidemiology of pneumonia and can stimulate and inform new studies.

The authors believe this is the only case–control study on the risk factors of CAP ever conducted in an unselected adult population in Africa. As the major risk factors were unknown at the outset, the study examined many different exposures simultaneously. Although this leads to a risk of false-positive associations, the purpose was to scan a broad range of potential risks that could be explored and confirmed in subsequent focused studies. As such, several key risk factors have been identified that are amenable to vaccination or to changes in lifestyle habits. Furthermore, in this setting the study has demonstrated that the risk factors for pneumonia among men and women were sufficiently different that they should be investigated and managed separately.

## Conclusion

This study was designed as a hypothesis-generating study. It has identified a number of potential risk factors that suggest that interventions that induce changes in lifestyle habits (especially smoking and the use of other drugs) may have a beneficial impact on the incidence of pneumonia in this population—these are worth considering in confirmatory studies. Previous history of pneumonia was the strongest risk factor of all in this study, suggesting a target for post-discharge vaccination.

## Additional file

Additional file 1: Supplementary information on the risk factors for pneumonia in Adults. (DOCX 142 kb)
