# Supplementary material for: Risk factors for community-acquired pneumonia among adults in Kenya: a case–control study
Source: Pneumonia (Nathan). 2017 Nov 25;9:17. doi: 10.1186/s41479-017-0041-2 (PMC5702239; doi:10.1186/s41479-017-0041-2)
Supplement: Additional file 1: — Supplementary information on the risk factors for pneumonia in Adults. (DOCX 142 kb) [file 41479_2017_41_MOESM1_ESM.docx]

RISK FACTORS FOR COMMUNITY ACQUIRED PNEUMONIA AMONG ADULTS IN KENYA: A CASE-CONTROL STUDY – **SUPPLEMENTARY INFORMATION**

**Authors:**

Muthumbi E^*1^, Lowe BS^1, 2^, Muyodi C^3^, Getambu E^3^, Gleeson F^4^, Scott JAG^1, 5^

**Institutions**

1. KEMRI-Wellcome Trust Research Programme. Center for Geographical Medicine Research Coast. Kenya
2. Centre for Tropical Medicine & Global Health, University of Oxford, UK
3. Coast Provincial General Hospital, Mombasa, Kenya
4. Department of Radiology, Churchill Hospital, University of Oxford, UK
5. Department of Infectious Disease Epidemiology, London School of Hygiene & Tropical Medicine, London, UK

*Corresponding author

Dr. Esther Muthumbi

KEMRI-Wellcome Trust Research Programme,

P.O BOX 230-80108,

Kilifi, KENYA.

[emuthumbi@kemri-wellcome.org](mailto:emuthumbi@kemri-wellcome.org)

**Table of contents**

1. S1: Power of the study
2. S2: Selected variables missing more than 10 observations and their association with case control status.
3. S3: Top ten most common presenting diagnosis among controls (*n* = 1202)
4. S4: Univariate analysis of clinical risk factors and risk of pneumonia
5. S5: Univariate analysis of socio-demographic factors and the risk of pneumonia
6. S6: Univariate analysis of exposure to sources of air pollution and the risk of pneumonia
7. S7: Univariate analysis of drug use and risk of pneumonia
8. S8: Univariate analysis of contact patterns and risk of pneumonia
   1. S8a: Crowding in the house and risk of pneumonia
   2. S8b: Contact with possible sources of infection and pneumonia
   3. S8c: Contact with animals and pneumonia
9. S9: Definition of select questionnaire variables

**Table S1: Power of the study**

| **Prevalence** | **Odds Ratio** | | | | |
| --- | --- | --- | --- | --- | --- |
|  | **1.5** | **2.0** | **3.0** | **5.0** | **10.0** |
| 0.02 | 20% | 50% | 89% | 99% | 100% |
| 0.06 | 41% | 86% | 100% | 100% | 100% |
| 0.10 | 56% | 96% | 100% | 100% | 100% |
| 0.14 | 67% | 99% | 100% | 100% | 100% |
| 0.18 | 74% | 99% | 100% | 100% | 100% |
| 0.22 | 79% | 100% | 100% | 100% | 100% |
| 0.25 | 82% | 100% | 100% | 100% | 100% |

**Table S2: Selected variables missing more than 10 observations and their association with case control status**

| Variable | Number present  *n* = 1483 | Missing in cases  *n* = 281 | Missing in controls  *n* = 1202 |
| --- | --- | --- | --- |
| HIV infection | 1456 | 0 (0) | 27 (0.02) |
| Sickle cell status | 1468 | 2 (0.01) | 13 (0.01) |
| Presence of BCG scar***** | 1424 | 27(0.10) | 32 (0.03) |
| Malaria | 1450 | 3 (0.01) | 30 (0.02) |
| Anemia | 1468 | 2 (0.01) | 13 (0.01) |
| HbA1C | 1393 | 12 (0.04) | 78 (0.06) |
| Splenomegaly | 1459 | 13(0.05) | 11 (0.01) |
| MUAC | 1461 | 11 (0.04) | 11 (0.01) |
| BMI***** | 1442 | 24 (0.09) | 17 (0.01) |
| ABO blood group | 1450 | 17 (0.06) | 16 (0.01) |

* Variables introduced later into the study

**Table S3: The ten most common presenting diagnosis among controls in rank order (*n* = 1202)**

| **Diagnosis** | **n** | **%** |
| --- | --- | --- |
| Malaria | 316 | 26% |
| Diarrhea | 267 | 22% |
| Upper respiratory tract infection | 122 | 10% |
| Chronic fever | 47 | 4% |
| Trauma | 43 | 4% |
| Anemia | 35 | 3% |
| Gastritis | 34 | 3% |
| Abscess and other skin infections | 30 | 2% |
| Other (not specified) | 30 | 2% |
| Surgical patients | 28 | 2% |

**Table S4: Univariate analysis of clinical risk factors and risk of pneumonia**

| **Variable** | | |  | **Control** | **%** | **Case** | **%** | **OR** | **95% CI** | **LR *p*-value** |
| --- | --- | --- | --- | --- | --- | --- | --- | --- | --- | --- |
| HIV infection | | No | | 819 | 69.7 | 134 | 47.7 | 1 |  |  |
|  | | Yes | | 356 | 30.3 | 147 | 52.3 | 2.82 | (2.11,3.78) | <0.001 |
| Chronic cough | | No | | 1181 | 98.3 | 269 | 96.8 | 1 |  |  |
|  | | Yes | | 21 | 1.7 | 9 | 3.2 | 2.02 | (0.90,4.54) | 0.102 |
| History of coryza | | No | | 919 | 76.5 | 158 | 56.8 | 1 |  |  |
|  | | Yes | | 283 | 23.5 | 120 | 43.2 | 2.74 | (2.06,3.65) | <0.001 |
| History of sore throat | | No | | 1127 | 93.8 | 252 | 90.6 | 1 |  |  |
|  | | Yes | | 74 | 6.2 | 26 | 9.4 | 1.66 | (1.03,2.67) | 0.037 |
| Presence of BCG scar | | No | | 140 | 12.0 | 47 | 18.5 | 1 |  |  |
|  | | Yes | | 1030 | 88.0 | 207 | 81.5 | 0.55 | (0.38,0.81) | 0.002 |
| Splenomegaly | | No | | 1110 | 93.2 | 222 | 82.8 | 1 |  |  |
|  | | Yes | | 81 | 6.8 | 46 | 17.2 | 2.88 | (1.94,4.28) | <0.001 |
| Malaria | | No | | 1017 | 86.8 | 269 | 96.8 | 1 |  |  |
|  | | Yes | | 155 | 13.2 | 9 | 3.2 | 0.23 | (0.11,0.45) | <0.001 |
| Anemia | | No | | 582 | 48.9 | 77 | 27.6 | 1 |  |  |
|  | | Yes | | 607 | 51.1 | 202 | 72.4 | 2.60 | (1.94,3.47) | <0.001 |
| Anti A (Blood group) | | No | | 378 | 31.9 | 86 | 32.6 | 1 |  |  |
|  | | Yes | | 808 | 68.1 | 178 | 67.4 | 0.97 | (0.72,1.29) | 0.809 |
| History of previous pneumonia | | | |  |  |  |  |  |  | <0.001 |
|  | No history | | | 1180 | 98.3 | 248 | 89.2 | 1 |  |  |
|  | > 2 yrs ago | | | 4 | 0.3 | 17 | 6.1 | 20.47 | (6.76,61.97) | |
|  | <2 yrs ago | | | 16 | 1.3 | 13 | 4.7 | 3.93 | (1.84,8.37) |  |
| History of previous TB | | | |  |  |  |  |  |  | 0.205 |
|  | No history | | | 1177 | 97.9 | 267 | 96.0 | 1 |  |  |
|  | > 2 yrs ago | | | 19 | 1.6 | 8 | 2.9 | 1.97 | (0.83,4.64) |  |
|  | <2 yrs ago | | | 6 | 0.5 | 3 | 1.1 | 2.11 | (0.52,8.62) |  |
| Sickle cell status |  | | |  |  |  |  |  |  | 0.700 |
|  | AA | | | 1073 | 90.3 | 251 | 89.9 | 1 |  |  |
|  | AS/ASF | | | 114 | 9.6 | 27 | 9.7 | 1.01 | (0.65, 1.58) | |
|  | SS | | | 1 | 0.1 | 1 | 0.4 | 3.43 | (0.21,55.9) |  |
|  |  | | |  |  |  |  |  |  |  |
| HbA1C | <4.0 | | | 677 | 60.2 | 141 | 52.4 | 0.7 | (0.53,0.93) | 0.027 |
|  | 4.0-5.6 | | | 397 | 35.3 | 118 | 43.4 | 1 |  |  |
|  | 5.7-6.4 | | | 29 | 2.6 | 3 | 1.1 | 0.34 | (0.10,1.15) |  |
|  | >6.5 | | | 21 | 1.9 | 7 | 2.6 | 1.11 | (0.46,2.71) |  |
|  |  | | |  |  |  |  |  |  |  |
| MUAC | <22 | | | 200 | 16.8 | 65 | 24.1 | 1 |  | <0.001 |
|  | 22-23 | | | 179 | 15 | 86 | 31.9 | 1.45 | (0.99,2.13) |  |
|  | 24-25 | | | 239 | 20.1 | 65 | 24.1 | 0.8 | (0.53,1.20) |  |
|  | 26-27 | | | 292 | 24.5 | 34 | 12.6 | 0.34 | (0.21,0.53) |  |
|  | 28+ | | | 281 | 23.6 | 20 | 7.4 | 0.21 | (0.12,0.35) |  |
| BMI | <18.5 | | | 423 | 35.7 | 131 | 50.9 | 1.82 | (1.38,2.39) | <0.001 |
|  | 18.5-24.9 | | | 709 | 59.8 | 123 | 47.9 | 1 |  |  |
|  | 25-29.9 | | | 43 | 3.6 | 2 | 0.8 | 0.27 | (0.06,1.13) |  |
|  | >29.9 | | | 10 | 0.9 | 1 | 0.4 | 0.6 | (0.10,0.25) |  |

**Table S5: Univariate analysis of socio-demographic factors and the risk of pneumonia**

|  | **Categories** | **Control** | **%** | **Case** | **%** | **OR** | **95%CI** | **LR *p*-value** |
| --- | --- | --- | --- | --- | --- | --- | --- | --- |
| Level of education | None | 221 | 18.4 | 72 | 25.9 | 1 |  | <0.001 |
|  | 1-6 years | 244 | 20.3 | 63 | 22.7 | 0.64 | (0.42, 0.97) | |
|  | Primary | 313 | 26.0 | 65 | 23.4 | 0.45 | (0.29, 0.70) | |
|  | Secondary | 310 | 25.8 | 64 | 23.0 | 0.43 | (0.27, 0.68) | |
|  | Tertiary | 114 | 9.5 | 14 | 5.0 | 0.27 | (0.14, 0.52) | |
|  |  |  |  |  |  |  |  |  |
| Employment Status | Unemployed | 423 | 35.2 | 86 | 30.9 | 1 |  | 0.002 |
|  | Employed | 715 | 59.5 | 187 | 67.3 | 1.34 | (0.97, 1.86) | |
|  | In Education | 64 | 5.3 | 5 | 1.8 | 0.35 | (0.13, 0.93) | |
|  |  |  |  |  |  |  |  |  |
| Marital status | Single | 359 | 29.9 | 79 | 28.3 | 1 |  | 0.009 |
|  | Married | 730 | 60.7 | 156 | 55.9 | 1.12 | (0.77, 1.63) | |
|  | Separated | 37 | 3.1 | 12 | 4.3 | 1.57 | (0.74, 3.33) | |
|  | Divorced | 23 | 1.9 | 14 | 5.0 | 3.44 | (1.59, 7.40) | |
|  | Widowed | 53 | 4.4 | 18 | 6.5 | 2.15 | (1.06, 4.35) | |
|  |  |  |  |  |  |  |  |  |
| Religion | Traditional | 67 | 5.6 | 25 | 9.0 | 1 |  | 0.006 |
|  | Muslim | 224 | 18.6 | 50 | 18.0 | 0.51 | (0.27, 0.96) | |
|  | Christian | 868 | 72.2 | 184 | 66.2 | 0.46 | (0.26, 0.82) | |
|  | Other | 43 | 3.6 | 19 | 6.8 | 1.00 | (0.46, 2.19) | |
|  |  |  |  |  |  |  |  |  |
| Income (Ksh) | No income | 263 | 21.9 | 53 | 19.1 | 1 |  | 0.521 |
|  | 1-1199 | 200 | 16.7 | 52 | 18.7 | 1.28 | (0.83, 1.99) | |
|  | 1200-1999 | 152 | 12.7 | 40 | 14.4 | 1.31 | (0.80, 2.13) | |
|  | 2000-2499 | 153 | 12.8 | 30 | 10.8 | 0.98 | (0.57, 1.67) | |
|  | 2500-2999 | 129 | 10.8 | 23 | 8.3 | 0.9 | (0.50, 1.61) | |
|  | 3000+ | 302 | 25.2 | 80 | 28.8 | 1.28 | (0.80, 2.04) | |
|  |  |  |  |  |  |  |  |  |
| Ethnic group | Giriama | 184 | 15.3 | 51 | 18.2 | 1 |  | 0.121 |
|  | Mijikenda | 233 | 19.4 | 45 | 16.0 | 0.67 | (0.42, 1.07) | |
|  | Taita | 70 | 5.8 | 25 | 8.9 | 1.28 | (0.70, 2.32) | |
|  | Kamba | 197 | 16.4 | 47 | 16.7 | 0.81 | (0.49, 1.35) | |
|  | Kikuyu | 86 | 7.2 | 20 | 7.1 | 0.76 | (0.41, 1.42) | |
|  | Luo | 164 | 13.6 | 45 | 16.0 | 0.9 | (0.54, 1.51) | |
|  | Luhya | 119 | 9.9 | 19 | 6.8 | 0.51 | (0.27, 0.96) | |
|  | Other | 149 | 12.4 | 29 | 10.3 | 0.66 | (0.38, 1.14) | |
|  |  |  |  |  |  |  |  |  |
| Roof type | Grass | 523 | 43.6 | 125 | 45.0 | 1 |  | 0.758 |
|  | Corrugated | 587 | 48.9 | 135 | 48.6 | 0.96 | (0.72, 1.27) | |
|  | Asbestos | 15 | 1.3 | 4 | 1.4 | 1.01 | (0.33, 3.15) | |
|  | Concrete | 57 | 4.8 | 9 | 3.2 | 0.65 | (0.31, 1.36) | |
|  | Tiled | 18 | 1.5 | 4 | 1.4 | 0.89 | (0.29, 2.70) | |
|  |  |  |  |  |  |  |  |  |

**Table S6: Univariate analysis of exposure to sources of air pollution and the risk of pneumonia**

| **Variable** |  | **Control** | **%** | **Case** | **%** | **OR** | **95% CI** | **LR *p*-value** |
| --- | --- | --- | --- | --- | --- | --- | --- | --- |
| Site of cooking | Outdoor | 154 | 12.9 | 33 | 11.9 | 1 |  |  |
|  | Indoors | 1039 | 87.1 | 244 | 88.1 | 1.08 | (0.72, 1.62) | 0.709 |
|  |  |  |  |  |  |  |  |  |
| Number of ventilations in cooking room | |  |  |  |  |  |  |  |
|  | 1 | 224 | 18.8 | 73 | 26.4 | 1 |  | 0.016 |
|  | >1 | 812 | 68.2 | 171 | 61.7 | 0.61 | (0.44, 0.85) |  |
|  | Outdoor | 154 | 12.9 | 33 | 11.9 | 0.65 | (0.41, 1.04) |  |
|  |  |  |  |  |  |  |  |  |
| Subject sleeps in the cooking room | No | 569 | 47.69 | 121 | 43.7 | 1 |  |  |
|  | Yes | 624 | 52.3 | 156 | 56.3 | 1.16 | (0.89, 1.52) | 0.274 |
|  |  |  |  |  |  |  |  |  |
| Type of fuel used | Gas/electricity | 41 | 3.4 | 7 | 2.5 | 1 |  | 0.069 |
|  | Charcoal | 232 | 19.5 | 39 | 14.1 | 1.76 | (0.74, 4.19) |  |
|  | Wood | 289 | 24.2 | 79 | 28.5 | 1 | (0.42, 2.41) |  |
|  | Kerosene | 630 | 52.9 | 152 | 54.9 | 1.42 | (0.62, 3.24) |  |
|  |  |  |  |  |  |  |  |  |
| Cooks for him/herself | No | 335 | 27.9 | 91 | 32.7 | 1 |  |  |
|  | Yes | 866 | 72.1 | 187 | 67.3 | 0.76 | (0.57, 0.99) | 0.050 |
|  |  |  |  |  |  |  |  |  |
| Use of mosquito coils | No | 743 | 62.6 | 174 | 63 | 1 |  |  |
|  | Yes | 443 | 37.4 | 102 | 37 | 0.97 | (0.74, 1.28) | 0.838 |
|  |  |  |  |  |  |  |  |  |
| Exposure to air conditioning | No | 1163 | 96.9 | 272 | 97.8 | 1 |  |  |
|  | Yes | 37 | 3.1 | 6 | 2.2 | 0.67 | (0.28, 1.61) | 0.346 |

**Table S7: Univariate analysis of drug use and risk of pneumonia**

| **Variable** |  | **Control** | **%** | **Cases** | **%** | **OR** | **95% CI** | **LR *p*-value** |
| --- | --- | --- | --- | --- | --- | --- | --- | --- |
| Alcohol | No | 984 | 81.9 | 194 | 69.8 | 1 |  |  |
|  | Yes | 218 | 18.1 | 84 | 30.2 | 2.08 | (1.51, 2.81) | <0.001 |
| Beer | No | 1077 | 89.7 | 225 | 80.9 | 1 |  |  |
|  | Yes | 124 | 10.3 | 53 | 19.1 | 2.05 | (1.41, 2.89) | <0.001 |
| Traditional brew | No | 1057 | 87.9 | 233 | 82.9 | 1 |  |  |
|  | Yes | 145 | 12.1 | 48 | 17.1 | 1.53 | (1.04, 2.17) | 0.030 |
| Busaa | No | 1187 | 98.8 | 272 | 97.8 | 1 |  |  |
|  | Yes | 14 | 1.2 | 6 | 2.2 | 2.02 | (0.76, 5.36) | 0.181 |
| Changaa | No | 1154 | 96.1 | 265 | 95.3 | 1 |  |  |
|  | Yes | 47 | 3.9 | 13 | 4.7 | 1.19 | (0.63, 2.25) | 0.609 |
| Matingas | No | 1198 | 99.8 | 275 | 98.9 | 1 |  |  |
|  | Yes | 3 | 0.2 | 3 | 1.1 | 3.57 | (0.71, 18.04) | 0.124 |
| Mnazi | No | 1088 | 90.6 | 239 | 86 | 1 |  |  |
|  | Yes | 113 | 9.4 | 39 | 14 | 1.62 | (1.06, 2.38) | 0.025 |
| Muratina | No | 1199 | 99.8 | 274 | 98.6 | 1 |  |  |
|  | Yes | 2 | 0.2 | 4 | 1.4 | 10.29 | (1.84, 57.66) | 0.007 |
| History of smoking | Never smoked | 874 | 72.7 | 148 | 53.2 | 1 |  |  |
|  | Ever smoked | 328 | 27.3 | 130 | 46.8 | 3.24 | (2.32, 4.53) | <0.001 |
| Current smoking habit | Non smoker | 697 | 58 | 117 | 42.1 | 1 |  |  |
|  | Passive smoker | 177 | 14.7 | 31 | 11.2 | 0.91 | (0.59, 1.42) | <0.001 |
|  | Ex-smoker | 88 | 7.3 | 31 | 11.2 | 2.93 | (1.79, 4.77) |  |
|  | Recent ex-smoker | 12 | 1 | 12 | 4.3 | 8.16 | (3.48, 19.14) |  |
|  | Current smoker | 228 | 19 | 87 | 31.2 | 3.08 | (2.13, 4.45) |  |
| Smoking pack years | 0 | 885 | 80.3 | 153 | 65.1 | 1 |  | <0.001 |
| (among current smokers) | 1-5 | 113 | 10.3 | 36 | 15.3 | 2.39 | (1.51, 3.81) |  |
|  | 6-10 | 50 | 4.5 | 22 | 9.4 | 3.35 | (1.87, 5.97) |  |
|  | 11-15 | 28 | 2.5 | 8 | 3.4 | 2.27 | (0.97, 5.29) |  |
|  | 16-20 | 11 | 1 | 11 | 4.7 | 7.82 | (3.19, 19.18) |  |
|  | 20+ | 15 | 1.4 | 5 | 2.1 | 2.83 | (0.97, 8.22) |  |
| Home rolled cigarettes | No | 1199 | 99.8 | 274 | 98.6 | 1 |  |  |
|  | Yes | 3 | 0.2 | 4 | 1.4 | 5.55 | (1.20, 25.72) | 0.028 |
| Miraa | No | 1160 | 96.5 | 247 | 88.8 | 1 |  |  |
|  | Yes | 42 | 3.5 | 31 | 11.2 | 3.36 | (2.05, 5.50) | <0.001 |
| Snuff | No | 1149 | 96 | 255 | 91.7 | 1 |  |  |
|  | Yes | 48 | 4 | 23 | 8.3 | 2.55 | (1.46, 4.44) | 0.001 |

CI, confidence interval; OR, odds ratio**Univariate analysis of contact patterns and risk of pneumonia**

**Table S8a: Crowding in the house and risk of pneumonia**

| **Variable** |  | **Control** | **%** | **Case** | **%** | **OR** | **95% CI** | **LR *p*-value** |
| --- | --- | --- | --- | --- | --- | --- | --- | --- |
| Number of other people in the house | 0 | 258 | 21.5 | 54 | 19.4 | 1 |  |  |
|  | 1 | 321 | 26.7 | 75 | 27.0 | 1.12 | (0.75, 1.66) | 0.031 |
|  | 2 | 169 | 14.1 | 46 | 16.5 | 1.33 | (0.83, 2.08) |  |
|  | 3 | 159 | 13.2 | 25 | 9.0 | 0.76 | (0.44, 1.26) |  |
|  | 4 | 112 | 9.3 | 26 | 9.4 | 1.09 | (0.64, 1.87) |  |
|  | 5–9 | 169 | 14.1 | 41 | 14.8 | 1.16 | (0.72, 1.86) |  |
|  | 10–30 | 14 | 1.2 | 11 | 4.0 | 3.97 | (1.68, 9.42) |  |
|  |  |  |  |  |  |  |  |  |
| Number of adults in the house | 0 | 285 | 24.5 | 64 | 25.2 | 1 |  |  |
|  | 1 | 607 | 52.2 | 124 | 48.8 | 0.91 | (0.64, 1.27) | 0.746 |
|  | 2 | 154 | 13.3 | 34 | 13.4 | 0.98 | (0.61, 1.57) |  |
|  | 3 | 63 | 5.4 | 17 | 6.7 | 1.21 | (0.65, 2.24) |  |
|  | 4+ | 53 | 4.6 | 15 | 5.9 | 1.28 | (0.67, 2.45) |  |
|  |  |  |  |  |  |  |  |  |
| Contact with children 5-14 in house | 0 | 788 | 67.6 | 176 | 69.0 | 1 |  |  |
|  | 1 | 150 | 12.9 | 37 | 14.5 | 1.09 | (0.72, 1.63) | 0.719 |
|  | 2 | 118 | 10.1 | 19 | 7.5 | 0.73 | (0.43, 1.23) |  |
|  | 3 | 59 | 5.1 | 13 | 5.1 | 0.94 | (0.50, 1.80) |  |
|  | 4+ | 50 | 4.3 | 10 | 3.9 | 0.84 | (0.40, 1.73) |  |
|  |  |  |  |  |  |  |  |  |
| Contact with under 5's in the house | 0 | 869 | 72.8 | 201 | 72.3 | 1 |  |  |
|  | 1 | 222 | 18.6 | 50 | 18.0 | 0.97 | (0.68, 1.38) | 0.262 |
|  | 2 | 84 | 7.0 | 18 | 6.5 | 0.92 | (0.54, 1.23) |  |
|  | 3 | 18 | 1.5 | 7 | 2.5 | 1.66 | (0.67, 4.10) |  |
|  | 4+ | 1 | 0.1 | 2 | 0.7 | 11.02 | (0.97, 124.7) | |
|  |  |  |  |  |  |  |  |  |
| Number of boys <5yrs. in home | 0 | 1003 | 83.8 | 223 | 79.9 | 1 |  |  |
|  | 1 | 167 | 14.0 | 44 | 15.8 | 1.22 | (0.84, 1.77) | 0.175 |
|  | 2 | 23 | 1.9 | 9 | 3.2 | 1.77 | (0.80, 3.92) |  |
|  | 3+ | 4 | 0.3 | 3 | 1.1 | 3.66 | (0.80, 16.75) | |
|  |  |  |  |  |  |  |  |  |
| Number of girls <5 yrs. in home | 0 | 1008 | 84.2 | 247 | 88.5 | 1 |  |  |
|  | 1 | 160 | 13.4 | 22 | 7.9 | 0.54 | (0.33, 0.87) | 0.038 |
|  | 2 | 24 | 2.0 | 8 | 2.9 | 1.28 | (0.56, 2.92) |  |
|  | 3+ | 5 | 0.4 | 2 | 0.7 | 1.82 | (0.35, 9.56) |  |
|  |  |  |  |  |  |  |  |  |
| Number of <5s in sleeping room | 0 | 897 | 75.3 | 213 | 76.9 | 1 |  |  |
|  | 1 | 201 | 16.9 | 47 | 17.0 | 0.96 | (0.66, 1.38) | 0.288 |
|  | 2 | 77 | 6.5 | 11 | 4.0 | 0.58 | (0.30, 1.13) |  |
|  | 3+ | 16 | 1.3 | 6 | 2.2 | 1.54 | (0.59, 4.07) |  |
|  |  |  |  |  |  |  |  |  |

CI, confidence interval; OR, odds ratio**Table S8b: Contact with possible sources of infection and pneumonia**

| **Variable** |  | **Control** | **%** | **Case** | **%** | **OR** | **95% CI** | **LR *p*-value** |
| --- | --- | --- | --- | --- | --- | --- | --- | --- |
| Contact with a boy aged <5y with coryza | No | 1179 | 99.0 | 272 | 98.2 | 1 |  |  |
|  | Yes | 12 | 1.0 | 5 | 1.8 | 1.73 | (0.59, 5.03) | 0.310 |
| Contact with a girl aged <5y with coryza | No | 1173 | 98.5 | 272 | 98.2 | 1 |  |  |
|  | Yes | 18 | 1.5 | 5 | 1.8 | 1.13 | (0.41, 3.14) | 0.810 |
| Exposure to a patient with chest infection | No | 1179 | 98.1 | 268 | 96.4 | 1 |  |  |
|  | Yes | 23 | 1.9 | 10 | 3.6 | 1.96 | (0.92, 4.20) | 0.097 |
| Lives with a bronchitic adult | No | 1191 | 99.3 | 275 | 98.9 | 1 |  |  |
|  | Yes | 9 | 0.8 | 3 | 1.1 | 1.45 | (0.39, 5.43) | 0.580 |
| Work and lives in different locations | No | 884 | 73.6 | 191 | 68.7 | 1 |  |  |
|  | Yes | 317 | 26.4 | 87 | 31.3 | 1.33 | (0.97, 1.81) | 0.073 |
| Visited a hospital in last 2 weeks | No | 992 | 84.8 | 219 | 85.6 | 1 |  |  |
|  | Yes | 178 | 15.2 | 37 | 14.5 | 1.00 | (0.68, 1.48) | 0.999 |
| Visited a disco in the last 2 weeks | No | 1165 | 97.0 | 275 | 98.9 | 1 |  |  |
|  | Yes | 36 | 3.0 | 3 | 1.1 | 0.34 | (0.10, 1.13) | 0.043 |
| Visited a bar in last 2 weeks | No | 808 | 67.3 | 165 | 59.4 | 1 |  |  |
|  | Yes | 393 | 32.7 | 113 | 40.1 | 1.47 | (1.09, 1.98) | 0.011 |
| Visited a café in last 2weeks | No | 1070 | 89.1 | 225 | 80.9 | 1 |  |  |
|  | Yes | 131 | 10.9 | 53 | 19.1 | 2.01 | (1.39, 2.89) | <0.001 |
| Visited a cinema in last 2 weeks | No | 1139 | 94.8 | 260 | 93.5 | 1 |  |  |
|  | Yes | 62 | 5.2 | 18 | 6.5 | 1.29 | (0.73, 2.24) | 0.386 |
| Used a matatu in last 2 weeks | No | 254 | 21.2 | 79 | 28.4 | 1 |  |  |
|  | Yes | 947 | 78.9 | 199 | 71.6 | 0.65 | (0.48,0.88) | 0.006 |
| Visited a church/mosque in last 2 weeks | No | 843 | 70.2 | 186 | 66.9 | 1 |  |  |
|  | Yes | 358 | 29.8 | 92 | 33.1 | 1.23 | (0.92, 1.64) | 0.166 |
|  |  |  |  |  |  |  |  |  |

CI, confidence interval; OR, odds ratio**Table S8c: Contact with animals and pneumonia**

| **Variable** |  | **Control** | **%** | **Case** | **%** | **OR** | **95% CI** | **LR *p*-value** |
| --- | --- | --- | --- | --- | --- | --- | --- | --- |
| Exposure to any animal | No | 683 | 56.8 | 114 | 41.0 | 1 |  |  |
|  | Yes | 519 | 43.2 | 164 | 59.0 | 2.03 | (1.54, 2.67) | <0.001 |
| Exposure to cats | No | 1116 | 92.8 | 255 | 91.7 | 1 |  |  |
|  | Yes | 86 | 7.2 | 23 | 8.3 | 1.16 | (0.71, 1.90) | 0.545 |
| Exposure to chickens | No | 731 | 60.8 | 135 | 48.6 | 1 |  |  |
|  | Yes | 471 | 39.2 | 143 | 51.4 | 1.78 | (1.36, 2.35) | <0.001 |
| Exposure to cows | No | 1154 | 96.0 | 260 | 93.5 | 1 |  |  |
|  | Yes | 48 | 4.0 | 18 | 6.5 | 1.77 | (1.00, 3.13) | 0.051 |
| Exposure to dogs | No | 1151 | 95.8 | 261 | 93.9 | 1 |  |  |
|  | Yes | 51 | 4.2 | 17 | 6.1 | 1.56 | (0.88, 2.79) | 0.131 |
| Exposure to ducks | No | 1190 | 99.0 | 272 | 96.8 | 1 |  |  |
|  | Yes | 12 | 1.0 | 9 | 3.2 | 3.51 | (1.44, 8.55) | 0.009 |
| Exposure to goats | No | 983 | 81.8 | 202 | 72.7 | 1 |  |  |
|  | Yes | 219 | 18.2 | 76 | 27.3 | 1.79 | (1.31, 2.45) | <0.001 |
| Exposure to sheep | No | 1185 | 98.6 | 271 | 97.5 | 1 |  |  |
|  | Yes | 17 | 1.4 | 7 | 2.5 | 1.8 | (0.73, 4.44) | 0.203 |
| Exposure to monkeys | No | 1199 | 99.8 | 273 | 98.2 | 1 |  |  |
|  | Yes | 3 | 0.2 | 5 | 1.8 | 7.3 | (1.70, 31.34) | 0.008 |
| Exposure to other animals | No | 1195 | 99.4 | 275 | 98.9 | 1 |  |  |
|  | Yes | 7 | 0.6 | 3 | 1.1 | 2.07 | (0.52, 8.21) | 0.299 |

CI, confidence interval; OR, odds ratio

**Table S9: Definition of select variables**

| **Variable** | **Definition** | **Questionnaire** |
| --- | --- | --- |
| Recent ex-smoker | Patients who stopped smoking at the same age they were when they were admitted at the hospital | How old were you when you gave up smoking? |
| Alcohol | Patients who consume any form of alcohol | Do you drink alcohol? |
| Traditional brews | Consumption of any of the traditional brews listed in the questionnaire | Which alcoholic drink do you take? Options were matingas, beer, mnazi, muratina, chang’aa, busaa [tick all that apply] |
| History of previous pneumonia | History of pneumonia categorized as within 2 years of current admission or prior. | Have you ever been admitted to the hospital with a chest infection or pneumonia which is not TB? If yes, in which year was this (use the last admission) |
| Chronic bronchitis | History of chronic cough | In the last year have you had a period of coughing every day which lasted for more than 3 months? |
| Passive smoking | Exposure to passive smoking | How many other people in your household smoke cigarettes? |
| Animal contact | Exposure to animals | Do you come into contact with any animals in your work regularly (i.e. pass within 2m of them at least once a week) |
